# Urinary Multi-Omics Profiling Reveals Systemic Molecular Alterations in Progressive External Ophthalmoplegia

**DOI:** 10.3390/ijms262311257

**Published:** 2025-11-21

**Authors:** Michela Cicchinelli, Guido Primiano, Francesca Canu, Jacopo Gervasoni, Aniello Primiano, Lavinia Santucci, Anna Percio, Viviana Greco, Chiara Leoni, Andrea Sabino, Michelangelo Ardito, Giuseppe Zampino, Serenella Servidei, Andrea Urbani, Federica Iavarone

**Affiliations:** 1 Dipartimento di Scienze Biotecnologiche di Base Cliniche Intensivologiche e Perioperatorie, Università Cattolica del Sacro Cuore, 00168 Rome, Italy; michela.cicchinelli@unicatt.it (M.C.); francesca.canu@guest.policlinicogemelli.it (F.C.); anna.percio@unicatt.it (A.P.); viviana.greco@unicatt.it (V.G.); andrea.urbani@policlinicogemelli.it (A.U.); 2 Fondazione Policlinico Universitario ‘Agostino Gemelli’ IRCCS, 00168 Rome, Italy; guidoalessandro.primiano@policlinicogemelli.it (G.P.); jacopo.gervasoni@policlinicogemelli.it (J.G.); aniello.primiano@policlinicogemelli.it (A.P.); lavinia.santucci@guest.policlinicogemelli.it (L.S.); chiara.leoni@policlinicogemelli.it (C.L.); andrea.sabino@unicatt.it (A.S.); giuseppe.zampino@unicatt.it (G.Z.); serenella.servidei@unicatt.it (S.S.); 3 Dipartimento di Neuroscienze, Università Cattolica del Sacro Cuore, 00168 Rome, Italy; 19.miche.97@gmail.com; 4 Dipartimento di Scienze della Vita e Sanità Pubblica, Università Cattolica del Sacro Cuore, 00168 Rome, Italy

**Keywords:** Progressive External Ophthalmoplegia (PEO), mitochondrial diseases, multi-omics, proteomics, metabolomics, LC-MS/MS, ATR-FTIR, extracellular matrix, immune response, mitochondrial dysfunction, molecular mechanisms, urine biomarkers, multi-omics integration

## Abstract

Advances in next-generation sequencing have significantly improved the molecular diagnosis of mitochondrial diseases (MDs), a group of heterogeneous neurogenetic disorders. However, progress in understanding their pathogenic mechanisms and translating this knowledge into effective therapies remains limited. Elucidating the molecular determinants of phenotypic variability in primary MDs is essential to uncover disease mechanisms and identify novel therapeutic targets. We investigated a cohort of eight adult patients with genetically confirmed Progressive External Ophthalmoplegia (PEO)—an extremely rare mitochondrial disorder—and compared them with eight age- and sex-matched healthy controls. A comprehensive multi-omics approach combining LC–MS/MS-based proteomics, UPLC–MS/MS-based metabolomics, ATR–FTIR spectroscopy, and chemometric multivariate analysis was employed to identify molecular alterations associated with mitochondrial dysfunction. Distinct proteomic and metabolic patterns related to energy metabolism were observed in PEO patients, correlating with their genetic background. Metabolomic analysis showed altered amino acid levels (seven statistically relevant) and disruptions in the metabolism of cysteine, methionine, and glutathione; proteomics finding (154 differentially expressed proteins) revealed dysregulation in extracellular matrix (ECM) organization and immune response pathways. This integrative analytical strategy offers new insights into the molecular complexity of PEO and mitochondrial disorders. The identification of disease-associated molecular signatures may enhance the understanding of pathogenic mechanisms and support the development of improved diagnostic and therapeutic approaches for MDs.

## 1. Introduction

Our understanding of mitochondria has significantly advanced beyond their traditional characterization as the cell’s “powerhouse”. These highly dynamic organelles are now seen as integral components of numerous cellular processes. Given their multifaceted functions, mitochondrial dysfunction has been increasingly associated with the onset and progression of a wide spectrum of diseases. Metabolically, mitochondria play a pivotal role, participating in essential biochemical pathways such as the tricarboxylic acid (TCA) cycle, oxidative phosphorylation (OxPhos), fatty acid oxidation (FAO), and amino acid metabolism [1,2,3].

Beyond generating energy through the ATP synthesis, mitochondria regulate calcium (Ca^2+^) homeostasis, initiate cell differentiation and apoptosis, and synthesize several biomolecules [4]. Mitochondrial metabolites, such as reactive oxygen species (ROS), act as mediators of cellular signaling, influencing several cellular processes. The interplay between energy metabolism and redox signaling within these organelles is essential for preserving homeostasis, ensuring a critical equilibrium necessary for cellular integrity and optimal function [5]. Disruption of this balance contributes to altered bioenergetic processes and redox signaling, which represent key pathogenic mechanisms in primary mitochondrial diseases (PMDs)—a clinically heterogeneous group of genetic disorders caused by defects in the mitochondrial respiratory chain [6]. Mitochondrial diseases predominantly affect tissues and organs with high energy demands, such as skeletal muscle, cardiac muscle, and the central nervous system. Clinical manifestations are highly variable, both in severity and age of onset, ranging from early childhood to late adulthood. These disorders may present with isolated organ involvement or, more commonly, with multisystemic manifestations [7]. Genetically, mitochondrial diseases are driven by pathogenic variants in either mitochondrial DNA (mtDNA) or nuclear DNA (nDNA), reflecting the dual genetic origin of proteins essential for mitochondrial structure and bioenergetic function. Both genomes encode critical components of the mitochondrial respiratory chain, and mutations in either can disrupt OxPhos, leading to cellular energy deficits and the wide clinical spectrum observed in these conditions [8].

Chronic Progressive External Ophthalmoplegia (CPEO or PEO) represents one of the most common clinical presentations within the spectrum of primary mitochondrial diseases (PMDs), particularly among the subgroup of Primary Mitochondrial Myopathies (PMM), a category of disorders marked by predominant skeletal muscle involvement resulting from impaired mitochondrial OxPhos [9]. PEO is one of the most frequent clinical presentations of PMDs, although overall it remains a rare disorder.

PEO is defined by bilateral ptosis and a slowly progressive; it may present as an isolated ocular myopathy, frequently occurs alongside more widespread skeletal muscle involvement or systemic features. The condition is genetically heterogeneous, resulting from pathogenic variants in mtDNA, most commonly single large-scale deletions, or nuclear genes critical for mitochondrial DNA replication and maintenance, such as *POLG*, *TWNK*, *DGUOK*, and *SLC25A4* [10]. Epidemiological data support the clinical prominence among mitochondrial disorders: in the Italian Collaborative Network, more than half of genetically confirmed mitochondrial disease patients exhibited ocular myopathy, and in the North American Mitochondrial Disease Consortium (NAMDC), approximately 20% of cases were diagnosed with PEO. These findings underscore PEO as a hallmark of PMM and a key clinical gateway to the diagnosis of mitochondrial diseases, reflecting both its diagnostic significance and phenotypic variability [11,12].

Despite significant progress in identifying pathogenic genetic variants, the molecular characterization of PEO and other PMDs remains far from complete. One of the central challenges in this domain is the weak correlation between genotype and phenotype, whereby identical genetic variants can lead to vastly different clinical outcomes. This phenotypic variability complicates efforts to establish predictive genetic models and hinders accurate prognosis and personalized therapeutic strategies. In this context, the situation is further complicated by the fact that disease manifestations often arise from combined defects across multiple pathways—such as OxPhos, mtDNA maintenance, nucleotide metabolism, and mitochondrial dynamics—rather than from a single, clearly defined lesion. This complexity underscores the multifactorial nature of mitochondrial disease, where factors such as heteroplasmy levels, tissue-specific thresholds, nuclear–mitochondrial interactions, and compensatory responses collectively shape the clinical phenotype. Their interplay drives marked inter- and intrafamilial variability, making it difficult to identify consistent pathogenic mechanisms and to develop reliable biomarkers for diagnosis, stratification, and therapeutic monitoring [13,14]. Addressing these challenges is essential not only for refining molecular diagnostics but also for advancing targeted, mechanism-based therapies capable of altering disease trajectories or alleviating clinical symptoms in affected individuals [15]. The rarity and genetic heterogeneity of these conditions further complicate large-scale investigations, underscoring the need for complementary approaches such as metabolomic and proteomic profiling. The proper use of biochemical tools is crucial to close the gap between the genetic outcomes and the pathophysiological evidence.

Proteomics and metabolomics have emerged as powerful tools in the study of PMDs and PMMs, offering valuable opportunities to unravel the complex molecular mechanisms underlying these disorders. These integrative “omics” approaches provide comprehensive, system-level insights into the dynamic networks of proteins and metabolites involved in mitochondrial dysfunction, thereby enhancing our understanding of disease pathogenesis, progression, and potential therapeutic targets [11]. Proteomics, which involves the large-scale, in-depth characterization of the proteome using advanced mass spectrometry (MS)-based technologies, is particularly instrumental for high-throughput identification and quantification of mitochondrial proteins [16]. It enables the detection of alterations in protein abundance, post-translational modifications, and protein–protein interactions, all of which are critical for elucidating the functional consequences of mitochondrial impairments in these neurogenetic disorders. Metabolomics serves as a vital complement to proteomics by enabling comprehensive analysis of small molecules and metabolites generated during cellular metabolic processes [17]. Given the mitochondria’s central role in bioenergetics, metabolomic profiling is particularly well suited to capturing the metabolic derangements characteristic of PMDs. State-of-the-art analytical techniques, including nuclear magnetic resonance (NMR) spectroscopy and mass spectrometry (MS) in conjunction with high-performance liquid chromatography (HPLC), facilitate the precise quantification of metabolites involved in key mitochondrial pathways such as the TCA cycle, OxPhos, FAO, amino acid metabolism, and redox homeostasis [18,19]. These platforms enable the identification of disease-specific metabolic signatures that reflect underlying mitochondrial dysfunction, thereby offering novel insights into pathogenic mechanisms and potential therapeutic targets. Recent technological advancements, particularly in Fourier-transform infrared (FTIR) spectroscopy, have further expanded the analytical toolkit for mitochondrial research. FTIR enables rapid, non-destructive characterization of biomolecules by detecting specific vibrational modes corresponding to distinct functional groups [20,21,22]. In the context of mitochondrial diseases, FTIR has proven valuable for identifying alterations in lipid composition, protein secondary structure, and overall metabolite profiles, thereby providing additional layers of biochemical information relevant to disease pathophysiology and progression [23].

Urine represents a highly advantageous biological specimen for omics-based investigations, owing to its ease of collection, non-invasive nature, and the relatively large volumes that can be obtained without discomfort to the patient [24,25,26]. Beyond its traditional use in urological studies, urine contains a diverse array of metabolites and proteins reflective of both systemic physiology and tissue-specific processes, making it a valuable source for exploring non-renal pathologies, including metabolic and mitochondrial disorders [25,26,27,28,29]. This systems-level approach holds promise for the discovery of reliable biomarkers and the refinement of diagnostic methodologies, while also laying the groundwork for the identification of novel therapeutic targets to be explored in future studies. Therefore, the aim of this study is to explore the potential of urine-based proteomics and metabolomic profiling as a non-invasive strategy to improve the molecular characterization and diagnostic accuracy of PMDs.

## 2. Results

This research starts from a cohort of eight adult patients (≥18 years of age) with a genetically confirmed PMD, exhibiting a clinical phenotype consistent with PEO, recruited in collaboration with the Neurophysiopathology Unit at the Fondazione Policlinico Universitario Agostino Gemelli IRCCS through the “AD MAIORA” project (PNRR-MR1-2022-12376346). The selected cohort involved patients with mtDNA Single Large Deletion (*n* = 4) and mtDNA Multiple Deletions (*n* = 4; 2 *POLG* and 2 *TWNK*). A group of eight controls (Ctrl) was also recruited, matched with the patients in terms of age and sex, and with no evidence of urinary tract or kidney pathologies. For each patient, the clinical, laboratory, and instrumental data collected were entered into an eCRF. The characteristics of the PEO patients and controls are summarized in Table 1.

The average age in the PEO cohort (51.4 ± 8.5) reflects the onset of the treated pathology, which typically occurs in adulthood. Four patients shared mtDNA Single Large Deletion (Sdel), while four shared mtDNA Multiple Deletions (Mdel) due to variants in the nuclear genes *TWNK* (*n* = 2) and *POLG* (*n* = 2). The table also highlights a difference in sex representation. Nevertheless, according to the literature, sex is not considered a confounding factor in mitochondrial diseases [30], thus it has not been considered relevant for further considerations.

Routine clinical analyses performed on the patients’ blood and urine samples were available, with a focus on urinalysis results. Only one patient showed a non-negative, yet still low, leukocyte esterase level, indicating a general absence of potential urinary tract infections in the cohort.

The patients’ hydration status can significantly influence the composition of urine. While creatinine correction is commonly employed as a normalization strategy to ensure comparability across samples, there are cases where variations in creatinine production and excretion may lead to an inaccurate baseline. Mitochondrial diseases have been noted as one such scenario, because creatinine levels might be affected in mitochondrial patients [31,32].

In this study, the creatinine level in the patients’ cohort resulted in 77.22% coefficient of variation percentage (CV%), and for this reason was excluded and replaced with other normalization strategies. For metabolomic analysis, urinary amino acid concentrations were normalized to specific gravity to correct for variations in hydration status. For proteomic analysis, total protein in each urine sample was measured by protein assay, and equal amounts were digested to produce peptide digests of uniform concentration. An equal portion of each digest was then analyzed.

A crucial consideration in the evaluation was the fact that some of patients were on medication that could not be discontinued before urine sample collection. Furthermore, the exclusion of patients based on drug usage was avoided due to the rarity of the diseases, the limited availability of patients, and the potential loss of statistical power in data analysis. For comprehensive transparency, clarification about medications used is provided in the Material and Methods section.

Proteomics, metabolomics, and Fourier-transform infrared (FTIR) spectroscopy analyses were conducted on urine samples.

### 2.1. Proteomic Results

**Figure 1:**
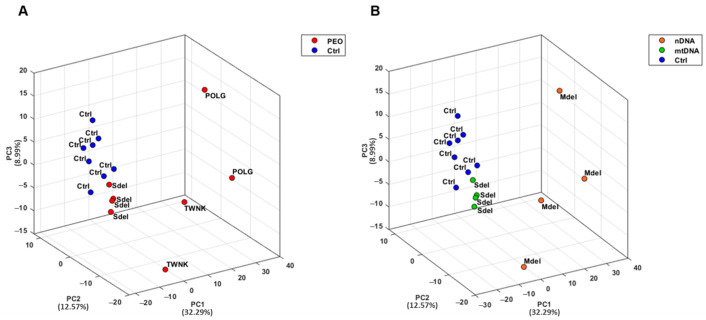
Scores plot of the PCA performed on PEO (*n* = 8) and Ctrl (*n* = 8) using the first three components. The percentage of explained variance was 32.29% (PC1), 12.57% (PC2), and 8.99% (PC3). (**A**) The scores plot shows a clear separation between PEO (red dots) and Ctrl (blue dots). The Ctrl exhibits high clustering, indicating greater inter-group similarity of the urinary proteome with respect to PEO. (**B**) PEO patients display heterogeneity, revealing a high degree of variability in terms of protein expression. Additionally, patients who tend to cluster together typically share the same genetic origin: nDNA variants for Mdel (*n* = 2 *POLG* and *n* = 2 *TWNK*, orange dots), and mtDNA variants for Sdel (*n* = 4, green dots). *POLG* (POLG); *TWNK* (TWNK); Single Large Deletion (Sdel); Multiple Deletions (Mdel).

Urine samples from both PEO patients and controls (Ctrl) were collected and analyzed in analytical triplicate using a mass spectrometry-based proteomic approach. A Principal Component Analysis (PCA) was conducted on proteomic data from PEO and Ctrl to explore similarities and differences between the two groups (Figure 1) [33]. PCA results demonstrated a strong clustering of the urinary proteome in Ctrl, with a clear separation from the PEO (Figure 1A). Notably, the PEO exhibited greater variability, showing a sub-group separation between Mdel and Sdel patients (Figure 1B).

**Figure 2:**
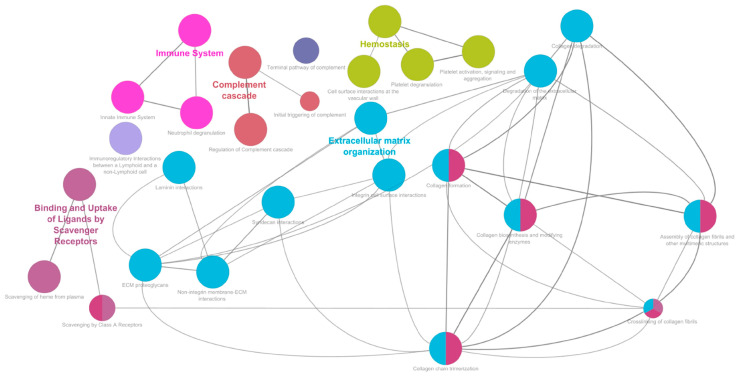
The pathways with the highest significance hierarchically belong to four major areas. Extracellular matrix organization (FDR 10^−14^), 54% of the identified proteins resulted differentially expressed; immune system (FDR 10^−14^), 61% of the identified proteins resulted differentially expressed; hemostasis (FDR 10^−14^), 56% of the identified proteins resulted differentially expressed; binding and uptake of ligands by scavenger receptors (FDR 10^−9^), 53% of the identified proteins resulted differentially expressed. All results were created with ClueGO v2.5.10.

A differential expression analysis and pathways enrichment were performed to compare PEO and Ctrl. The differential expression analysis was conducted using the PEO vs. Ctrl Fold Change (FC) values with threshold 1.5, and a statistical significance determined through three *p*-value thresholds (*p* < 0.1 (*), *p* < 0.05 (**), and *p* < 0.01 (***)). A total of 406 differentially expressed proteins were identified, of which 154 with statistical significance. The pathways enrichment analysis was conducted on the dataset using Reactome Pathways as the ontology source. Only pathways with an FDR < 0.05 were considered and selected for further analysis. The pathways with the highest statistical significance hierarchically belong to four major areas relative to the “Extracellular matrix organization”, “Immune System”, “Hemostasis”, and “Binding and uptake of ligands by scavenger receptors” macro-pathways (Supplementary Table S1). Matching these results with the differential expression analysis revealed that a high number of differentially expressed proteins characterized the enriched macro-pathways (Figure 2).

**Figure 3:**
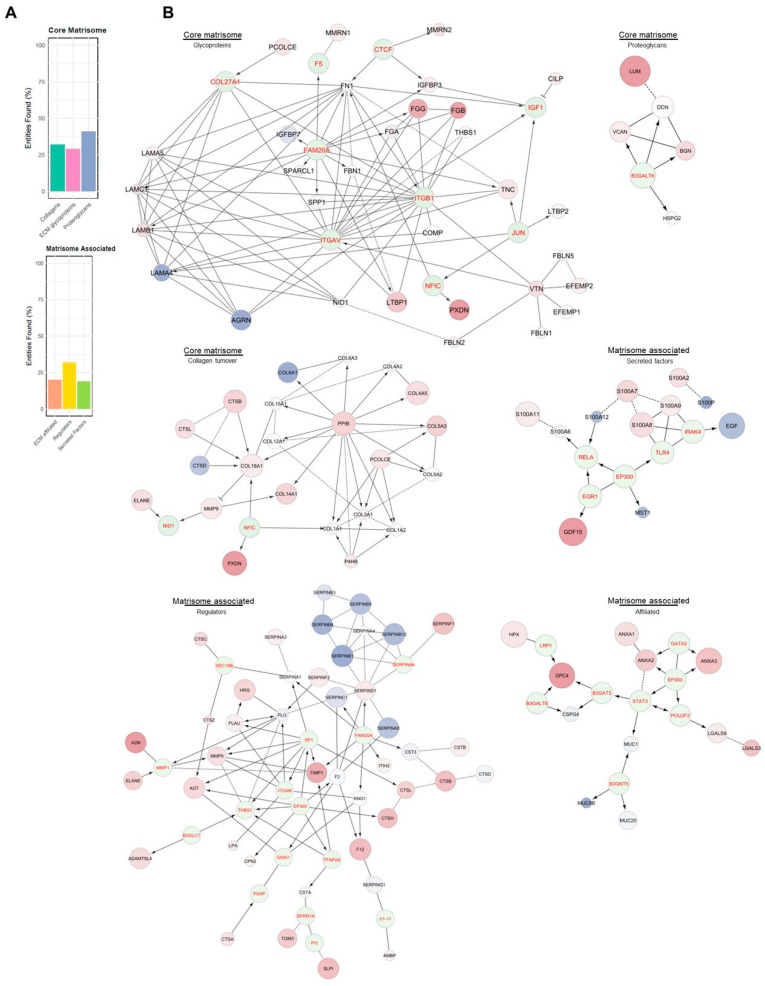
Impairments in matrisome mechanisms. (**A**) The percentage of the matrisome, categorized by groups, covered by experimental results. (**B**) Protein–protein interaction networks illustrate dysregulation within the matrisome and matrisome-associated proteins. A Fold Change (FC) threshold of 1.5 was applied, while the strength of the statistical significance was determined according to the *t*-test (*p*-value cutoff at 0.1, 0.05 and 0.01). Protein expression is reported as PEO (*n* = 8) vs. Ctrl (*n* = 8) ratio FC, with proteins showing FC > 1.5 in red and those with FC < −1.5 in blue. Statistical significance for PEO vs. Ctrl is displayed using different sizes for dots, with greater size for smaller *p*-values. The arrows indicate directional interactions, while the dotted lines represent indirect interactions. The labels shown correspond to gene names. The graphs reveal a general deregulation of structural ECM and a widespread impairment of proteins interacting with the ECM across the pathological state.

**Figure 4:**
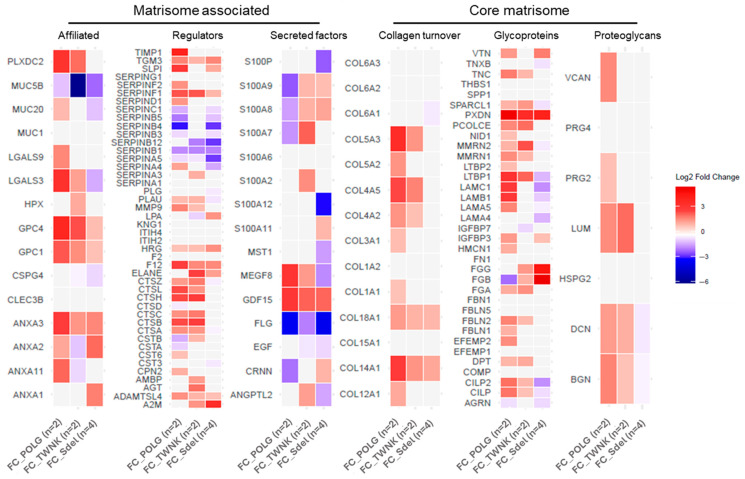
Heatmaps display the Log_2_FC values (*x*-axis) for the *POLG* vs. Ctrl, *TWNK* vs. Ctrl and Sdel vs. Ctrl ratios. A Fold Change (FC) threshold of 1.5 was applied. Proteins upregulated in the variants respect to Ctrl are shown in red, while the downregulated ones are in blue. Both *POLG* and *TWNK* exhibit overexpression of structural ECM components and many matrisome-associated enzymes respect to Ctrl. In contrast, Sdel shows no notable imbalance in the structural ECM components identified, and a matrisome-associated enzymes dysregulation is less pronounced than in the nuclear variants respect to Ctrl.

The enrichment of the “Extracellular matrix organization” sub-pathways (Supplementary Table S1) highlighted the involvement of the major mechanisms of ECM. Based on this evidence, it was necessary to evaluate the potential impairment in ECM mechanisms. To this end, proteomics data were compared with the MatrisomeDB 2.0 protein database [34]. The percentage of the matrisome covered by the experimental proteomic results (Figure 3A) described a dysregulated condition across all the matrisome categories in PEO (Figure 3B). For every category, the single contribution of the variants vs. Ctrl was also investigated. The differences in proteins expression between the variants and Ctrl were assigned using the FC values (Figure 4). In addition to the matrisome proteins displayed, CCN family member 2 (CCN2; FC 1.81) and TGF-beta receptor type-1 (TGFBR1; FC 2.03: (*)) were upregulated.

A detailed observation of the pathways responsible for “Immune system” enrichment revealed the presence of both innate and adaptive response, through the “Complement cascade” (FDR 10^−13^), the “Neutrophil degranulation” (FDR 10^−14^), and the “Immunoregulatory interactions between a lymphoid and a non-lymphoid cell” (FDR 10^−7^) sub-pathways. “Immunoregulatory interactions between a lymphoid and a non-lymphoid cell” pathway showed more than 50% of identified proteins overexpressed in patients. Intercellular adhesion molecule 1 (ICAM1; FC 3.20; (**)) was upregulated with high statistical significance. Among all the proteins identified in the “Neutrophil degranulation” pathway, those with a direct involvement in the process were investigated. Many of them showed upregulation in PEO, like Azurocidin (AZU1; FC 3.82), Neutrophil elastase (ELANE; FC 2.47), Myeloperoxidase (MPO; FC 16.13; (***)).

The other pathway of relevance in the context of the innate immune system is the “Complement cascade”, where most of the enzymes and regulators of the cascade were dysregulated in the pathological state. Particularly relevant is the upregulation of the C4 subunits C4A (FC 40.30; (***)) and C4B (FC −7.83; (***)), and C8B (FC 4.30; (*)), and C9 (FC 2.64; (**)), subunits of the membrane attack complex (MAC) which is involved in the destruction of target cells.

The *POLG*, *TWNK*, and Sdel variants’ contribution was also considered. All variants showed dysregulation of the complement cascade compared to Ctrl. Data from patients carrying variants, *POLG* and *TWNK*, did not display notable alterations in the regulatory enzymes, in contrast to Sdel which exhibited altered expression in most of them (Supplementary Figure S1).

**Figure 5:**
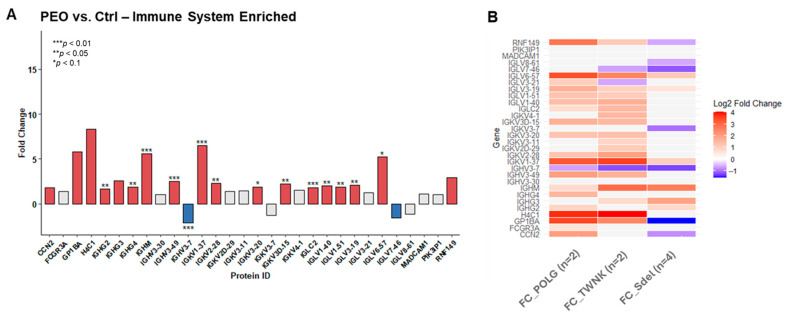
Expression of immune-related proteins in PEO. (**A**) The bar plot shows the protein expression of immune-specific proteins in PEO (*n* = 8) vs. Ctrl (*n* = 8). A Fold Change (FC) threshold of 1.5 was applied, while the strength of the statistical significance was determined according to the *t*-test (*p*-value cutoff at 0.1, 0.05 and 0.01). Protein expression is reported as PEO vs. Ctrl ratio FC, with proteins showing FC > 1.5 in red and those with FC < −1.5 in blue. Statistical significance for PEO vs. Ctrl is displayed using (*) for *p*-value < 0.1, (**) for *p*-value < 0.05, and (***) for *p*-value < 0.01. A total of 30 immune-specific proteins, expressed exclusively or predominantly within the immune system cells, are reported. Most of them show statistically significant upregulation in the pathological state. With proteins identified across granulocytes, monocytes, T cells, and B cells, both the innate and adaptive immune systems are involved. (**B**) The heatmap displays the Log_2_FC values (*x*-axis) for the *POLG* vs. Ctrl, *TWNK* vs. Ctrl, and Sdel vs. Ctrl ratios related to immune-specific proteins. A Fold Change (FC) threshold of 1.5 was applied. Proteins upregulated in the variants with respect to Ctrl are shown in red, while the downregulated ones are in blue.

Among the proteins identified within immune system pathways, many are not cell-specific. To assess the actual increase in immune-related proteins expression in PEO, the proteomic dataset was cross-referenced with genes classified as “*enriched*” in the Human Protein Atlas database for immune cells (the human immune cells—The Human Protein Atlas). This matching process identified 30 immune-specific proteins, expressed exclusively or predominantly within the immune system cells (Figure 5A). Most of them revealed statistically significant upregulation in the pathological state. The *POLG*, *TWNK*, and Sdel variants’ contribution is displayed (Figure 5B). The overexpression is associated with the nuclear variants *POLG* and *TWNK*, while Sdel shows a less compromised pattern.

The “Platelet degranulation” (FDR 10^−12^) and the “Cell surface interactions at the vascular wall” (FDR 10^−9^) emerged as the key pathways driving the “Hemostasis” enrichment. Several proteins directly related to the two mechanisms resulted differentially expressed. Proteins directly involved in the platelet degradation were upregulated, such as Fibrinogen alpha chain (FGA; FC 1.62), Fibrinogen beta chain (FGB; FC 15.85), Fibrinogen gamma chain (FGG; FC 12.21), and Multimerin-1 (MMRN1; FC 1.67). Among the proteins interacting with the vascular wall, Tyrosine-protein phosphatase non-receptor type substrate 1 (SIRPA; FC 1.56), Junctional adhesion molecule A (JAM-A; FC 1.99), and Junctional adhesion molecule C (JAM3; FC 5.29; (**)) showed upregulation.

The “Binding and Uptake of Ligands by Scavenger Receptors” macro-pathway showed 60% of the associated proteins, both directly and indirectly involved, upregulated in the pathological condition. In addition, Cytochrome c (CYCS; FC 5.49; (**)) and growth/differentiation factor 15 (GDF15; FC 5.00; (***)) were also upregulated. These findings may point out the condition of oxidative stress. Observing the pathways’ enrichment results considering this evidence, the ROS detoxification pathway (FDR 10^−4^) was highlighted. Proteins known to be indicators of stress and antioxidant response enzymes were investigated, revealing a widespread overexpression in PEO patients. Among them, Thioredoxin (TXN; FC 1.96), Peroxiredoxin-6 (PRDX6; FC 6.08; (**)), Peroxiredoxin-2 (PRDX2; FC 29.06); (***)), and various heat shock proteins like heat shock cognate 71 (HSPA8; FC 2.94; (*)), heat shock protein beta-1 (HSPB1; FC 1.95), heat shock protein HSP 90-alpha (HSP90AA1; FC 2.26), and heat shock 70 kDa protein 1B (HSPA1B; FC 2.66). In addition, the protein expression in *POLG*, *TWNK*, and Sdel patients were explored, showing for Sdel a less compromised spectrum in antioxidant and stress response enzymes compared to what has been documented in individuals with variants in nuclear genes (Supplementary Figure S2).

**Figure 6:**
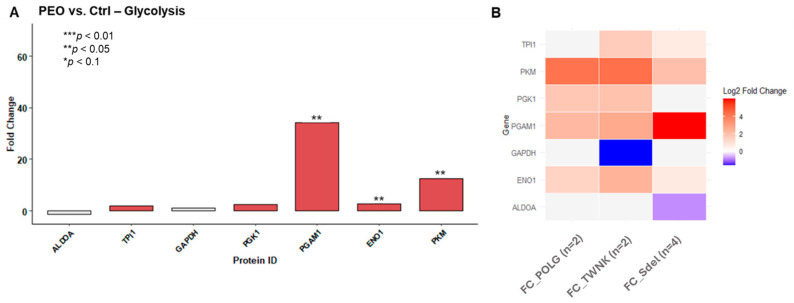
Glycolysis imbalance in PEO. (**A**) The bar plot shows the protein expression of the glycolysis pathway in PEO (*n* = 8) vs. Ctrl (*n* = 8). A Fold Change (FC) threshold of 1.5 was applied, while the strength of the statistical significance was determined according to the *t*-test (*p*-value cutoff at 0.1, 0.05, and 0.01). Protein expression is reported as PEO vs. Ctrl ratio FC, with proteins showing FC > 1.5 in red and those with FC < −1.5 in blue. Statistical significance for PEO vs. Ctrl is displayed using (*) for *p*-value < 0.1, (**) for *p*-value < 0.05, and (***) for *p*-value < 0.01. Several glycolytic enzymes resulted upregulated in the PEO group. (**B**) The heatmap displays the Log_2_FC values (*x*-axis) for the *POLG* vs. Ctrl, *TWNK* vs. Ctrl, and Sdel vs. Ctrl ratios related to glycolysis enzymes. A Fold Change (FC) threshold of 1.5 was applied. Proteins upregulated in the variants with respect to Ctrl are shown in red, while the downregulated ones are in blue. The most significant impairment appears to affect *TWNK*, followed by *POLG*. In the Sdel variant, alterations in PGAM1 and PKM are observed, but the overall condition is switched off compared to the nuclear variants.

Based on the results of the FTIR analysis (Figure 6A), glucose metabolism was further investigated. Correspondingly, differences in protein expression between patients carrying pathogenic variants and healthy controls were also observed in this context (Figure 6B).

### 2.2. Metabolomic Results

**Figure 7:**
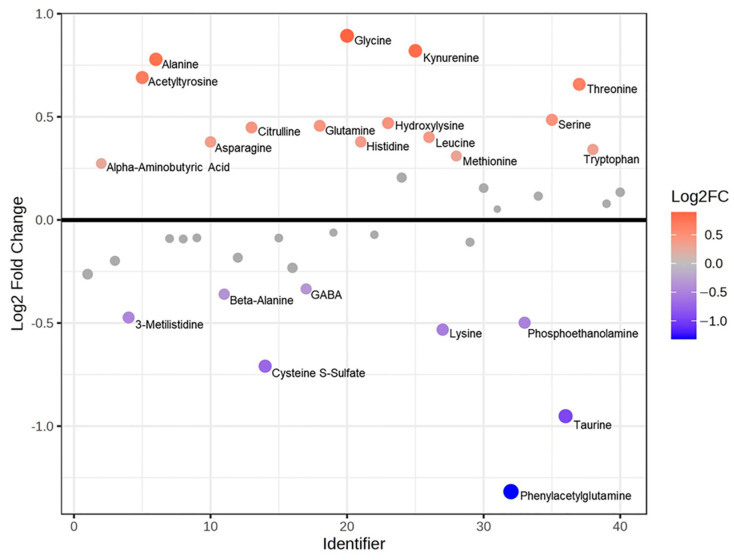
Fold Change ratio of amino acids between PEO (*n* = 8) and Ctrl (*n* = 8). Log2(FC) values for each amino acid are shown on the *y*-axis. An FC threshold of 1.2 and *t*-test based *p*-value set at 0.05 were used. Metabolites in red are increased in PEO compared to Ctrl, those in blue are decreased, and grey ones do not exceed the Fold Change threshold.

Metabolomic analyses, including mass spectrometry and Fourier-transform infrared (FTIR) spectroscopy, were conducted on urine samples to complement and support the proteomic findings. Mass spectrometry was employed to assess alterations in amino acid levels in PEO compared to Ctrl. FTIR spectroscopy was used to characterize the overall chemical composition of urine, with a particular focus on identifying metabolic disturbances associated with the disease state, especially those related to energy metabolism. The amino acids (AA) quantification was obtained through an LC–mass spectrometry-based analysis of urine samples. An integrative analysis (Joint Pathways Analysis) was executed using metabolomic and proteomic data and their FC values between PEO vs. Ctrl at pathway level (Table 2). The analysis was based on KEGG “human metabolic pathways”, with the aim to highlight the enriched pathways and the quantitative changes in the pathological state. Then, the single metabolomics’ contribution was evaluated through FC ratios (Figure 7) and *t*-test. Leucine (*p*-value 0.01), threonine (*p*-value 0.02), alanine (*p*-value 0.02), serine (*p*-value 0.03), and acetyltyrosine (*p*-value 0.04) were highlighted as significant and higher in PEO respect to Ctrl, while cysteine S-sulfate (*p*-value 0.04) and taurine (*p*-value 0.03) resulted in significant but lower PEO with respect to Ctrl.

**Figure 8:**
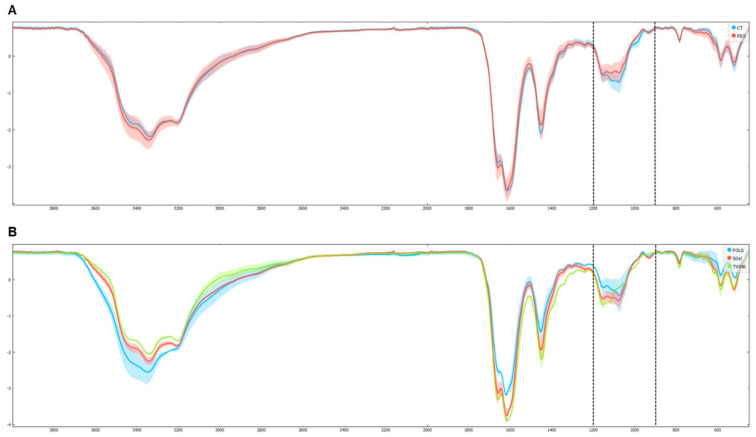
FTIR analysis results. (**A**) The average transmittance spectrum of PEO (*n* = 8) and Ctrl (*n* = 8) in the 4000–450 cm^−1^ wavenumber range. (**B**) The average transmittance spectra of POLG (*n* = 2), TWNK (*n* = 2), and Sdel (*n* = 4) in the 4000–450 cm^−1^ wavenumber range. The 1200–900 cm^−1^ is characterized by an evident change in transmittance. Images from Orange 3-3.35.0.

The FTIR analysis was conducted using an ATR-FTIR spectrophotometer [35]. All the spectra were recorded in the 4000–450 cm^−1^ wavenumber range in transmittance mode (T%). The average transmittance spectra of PEO and Ctrl (which contain the relative peaks of biomolecules such as proteins, lipids, carbohydrates and nucleic acids) were overlapped to explore the differences (Figure 8A). Then, the average transmittance spectra of the variants were also overlapped (Figure 8B) to show the changes between the three groups. Spectra were first analyzed in the functional group region (4000–1800 cm^−1^) and then in the fingerprint spectral region (1800–450 cm^−1^), revealing spectral variations. Specifically, differences in the area of carbohydrates (1200–800 cm^−1^) were highlighted in both PEO vs. Ctrl and *POLG*, *TWNK*, and Sdel variants comparisons. Subsequently, targeted analyses were conducted in this region to verify statistical significance and differential absorbance levels (*p*-value < 0.05, FC > |1.5|). The analysis showed several significant bands (region 1011–967 cm^−1^), with a lower absorbance in the PEO group with respect to Ctrl (Supplementary Figure S3), that can be allocated to the stretching vibrations of the C-O/C-C groups [36].

## 3. Discussion

The PCA analysis revealed a strong clustering of the urinary proteome in controls, clearly distinct from that of PEO patients. In contrast, the PEO group displayed greater variability, including a noticeable subgroup separation between individuals with Mdel and Sdel.

Specifically, the separation between patients with Mdel and those with a Sdel may reflect different molecular mechanisms or pathophysiological pathways contributing to disease variability [37]. These findings underscore the value of urinary proteomics as a non-invasive tool for stratifying patients and exploring mitochondrial disease subtypes. The differential expression and pathway enrichment analyses revealed key insights into the molecular alterations associated with PEO. The identification of 406 differentially expressed proteins, 154 of which reached statistical significance, underscores the extensive proteomic remodeling occurring in the disease state. The pathway enrichment analysis, focused on high-confidence pathways (FDR < 0.05), identified four major functional categories: “Extracellular Matrix Organization,” “Immune System,” “Hemostasis,” and “Binding and Uptake of Ligands by Scavenger Receptors.” These findings suggest that PEO is associated with disruptions in structural tissue integrity (as previously observed in murine models with mutations in TWNK [38]), immune responses (as shown in other MDs [39]), coagulation processes, and mechanisms of cellular uptake and clearance. The strong overlap between significantly altered proteins and the components of these enriched pathways indicates a biologically coherent signature, further supporting their potential relevance to PEO pathogenesis. This integrative approach provides a functional framework to better understand the systemic impact of mitochondrial dysfunction and may help identify novel biomarkers or therapeutic targets.

The “matrisome” offers comprehensive information on ECM and ECM-associated genes [40]. These proteins are divided into two major groups: the core matrisome, which consists of the structural components of the ECM (glycoproteins, collagens, and proteoglycans), and the matrisome-associated proteins, which play key roles in ECM organization, regulation, and cellular interactions. The matrisome-associated proteins are subdivided into ECM-affiliated proteins (which interact with the ECM in various ways), ECM regulators (enzymes that control ECM turnover), and secreted factors (proteins secreted by cells that modulate ECM functions or ECM-related cellular processes). Proteomic results (Figure 3A) displayed a dysregulated condition across all the matrisome categories in PEO (Figure 3B), suggesting both a structural impairment due to the upregulation of core matrisome components, and a functional impairment, considering the altered expression of enzymes regulating ECM mechanisms and their interactors.

The enrichment of “Extracellular Matrix Organization” sub-pathways points to a significant involvement of ECM-related processes in PEO pathophysiology. To further explore this, the proteomic dataset was mapped against MatrisomeDB 2.0, revealing broad dysregulation across all major matrisome categories, including core matrisome components (collagens, ECM glycoproteins, and proteoglycans) and matrisome-associated proteins. This suggests that the structural and regulatory integrity of the ECM is substantially altered in PEO.

Notably, the analysis of Fold Change (FC) values across variants relative to controls showed distinct expression patterns, emphasizing the molecular heterogeneity within the PEO group. Among the significantly upregulated proteins were CCN2 (FC 1.81), a known regulator of fibrosis and tissue remodeling [41], and TGFBR1 (FC 2.03, *p* < 0.1), a key component of the TGF-β signaling pathway, which is critically involved in ECM production and homeostasis [41,42]. These findings support the hypothesis that altered ECM dynamics—potentially driven by dysregulated TGF-β signaling—may play a pivotal role in the disease process and contribute to tissue-specific manifestations of PEO.

This comprehensive pathway and protein-level analysis highlight a multisystem dysregulation in PEO, particularly involving immune system activity, hemostasis, oxidative stress, and glucose metabolism.

For the Immune System Activation, a strong enrichment of immune-related pathways—such as complement cascade, neutrophil degranulation, and immunoregulatory interactions—suggests activation of both innate and adaptive immune responses in PEO [43,44]. The consistent upregulation of key effector proteins (e.g., MPO, ELANE, and ICAM1) reflects an inflammatory milieu, possibly contributing to tissue damage [45,46,47]. The especially high upregulation of C4A (FC 40.30) and other membrane attack complex (MAC) components indicates a potentially damaging activation of complements in these patients [48]. Cross-referencing with the Human Protein Atlas further confirmed that many of the differentially expressed proteins are immune cell-specific, validating a real increase in immune activity. Interestingly, POLG and TWNK variants show more pronounced immune activation than the mtDNA Sdel variant, hinting at genotype-specific immune involvement.

In chronically inflamed tissues, the abnormal expression of the ECM and its structural components, resulting from tissue remodeling processes, can influence the activation and survival of immune cells, thereby actively contributing to site-specific immune responses [49]. In tissues characterized by chronic inflammation, cytokines (such as TGF-β) and proteases such as matrix metalloproteinases can modify the ECM through the selective cleavage of its proteins and their receptors [50,51]. This leads to the generation of bioactive fragments that influence the infiltration, activity, and function of immune cells in inflamed tissues.

Evidence from recent studies supports a bidirectional relationship between mitochondrial dysfunction and immune/extracellular-matrix (ECM) activation. Mitochondrial damage promotes release of mitochondrial DAMPs (e.g., mtDNA, cardiolipin, and N-formyl peptides), and increased mitochondrial ROS and metabolic stress, all of which can activate innate immune receptors, inflammasomes, and cytokine production and thereby trigger downstream ECM remodeling and fibrotic responses [52]. Conversely, changes in ECM composition, stiffness, or cell-matrix signaling can perturb mitochondrial homeostasis and bioenergetics, creating a feed-forward loop in which ECM alterations exacerbate mitochondrial stress [53]. Taken together, the preponderance of evidence indicates that immune/ECM activation in conditions such as PEO is frequently secondary to primary mitochondrial dysfunction but once established, it may become self-sustaining and contribute directly to disease progression. Pathways such as Platelet Degranulation and Cell Surface Interactions at the Vascular Wall were enriched, with multiple Fibrinogen chains and adhesion molecules upregulated. This could suggest a pro-thrombotic or endothelial-activating environment in PEO patients, which might have clinical relevance in terms of vascular integrity. It also supports what has already been observed in the context of inflammation, with increased expression of ICAM1, which is responsible for the trans-endothelial migration of leukocytes.

For oxidative stress and mitochondrial dysfunction, the upregulation of scavenger receptor pathways, along with markers of oxidative stress such as GDF15 and CYCS, points toward mitochondrial distress and a sustained cellular stress response. Consistent overexpression of detoxifying and stress response proteins (e.g., PRDX2, TXN, and HSPs) further supports the presence of chronic oxidative imbalance in PEO. The more pronounced stress response in POLG and TWNK compared to Sdel again suggests a more severe mitochondrial compromise in nuclear-encoded variants.

Metabolomic findings appear to support the proteomic results, showing the presence of oxidative stress. PEO is characterized by altered amino acid levels, such as serine (implicated in the trans-sulfuration pathway and a precursor of cystathionine) and disruptions in the metabolism of cysteine, methionine, and glutathione. These alterations are confirmed by both differential and enrichment analyses. Additionally, a downregulation of cysteine-derived metabolites (sulfur-containing amino acids such as cystine, taurine, and sulfocysteine) is observed. Cysteine plays a key role in metabolic remodeling through various mechanisms: it contributes to redox regulation as a component of glutathione (a major antioxidant scavenger) [54,55], and is involved in protein S-cysteinylation. Moreover, cysteine can serve as a substrate for hydrogen sulfide (H_2_S) production, which supports mitochondrial electron transport chain activity and mediates the persulfidation of ATPase and glycolytic enzymes [56]. Cysteine catabolism also leads to the formation of other organic compounds, crucial for carbon and energy metabolism, such as pyruvate, which can then be converted to acetyl-CoA and enter the TCA cycle, or be utilized in the synthesis of fatty acids and α-ketoglutarate—a precursor of glutamate and also a key TCA cycle intermediate [56].

Proteomic data further reveal alterations in glucose metabolism, which are supported by FTIR results showing significant differences in absorbance in the carbohydrate region (1200–800 cm^−1^) in PEO, particularly across different variants, thereby adding another layer of metabolic dysregulation. This observation aligns with the overall hypothesis of energetic imbalance in PEO and supports the involvement of systemic metabolic remodeling.

Urine is a dynamic and integrative biofluid that captures signals from both renal function and systemic metabolism. The proteins and metabolites identified in our study likely arise from multiple biological sources, including glomerular filtration of plasma components, active tubular secretion, and, in some cases, cellular leakage linked to mitochondrial dysfunction and oxidative stress. In PEO, defective mitochondrial respiration can lead to increased production of reactive oxygen species, impaired energy metabolism, and secondary tissue damage in metabolically active organs such as skeletal muscle, liver, and kidney. Consequently, the altered urinary proteomic and metabolomic signatures observed may reflect systemic metabolic disturbances rather than solely renal processes. Therefore, urinary profiles in PEO may serve as non-invasive indicators of the multisystemic nature of the disease, providing insight into how mitochondrial dysfunction manifests at the organismal level.

Although the proteomic analysis highlighted several findings of particular interest, these results require future experimental validation to confirm their biological relevance. In particular, the increased expression of GDF15, already known for its association with mitochondrial stress. Similarly, the modulation of cytochrome c (CYCS), if confirmed, could support the hypothesis of a pro-inflammatory environment. The upregulation of CCN2 also could be validated to support the activation of TGF-β–related pathways and their involvement in tissue remodeling. Finally, proteins implicated in oxidative stress, such as PRDX2, require additional analyses to consolidate their contribution to the observed pathophysiological mechanisms.

## 4. Materials and Methods

### 4.1. Patients

All patients underwent comprehensive clinical evaluation by a neurologist specialized in mitochondrial medicine and were subsequently classified according to their clinical phenotype and underlying molecular diagnosis. All patients were receiving Coenzyme Q10 supplementation, except for two individuals, who were not taking any medications. In addition to Coenzyme Q10, one patient was also undergoing treatment with riboflavin.

### 4.2. Sample Collection

Urine samples were collected after a 12 h fasting period and abstention from medication intake, following the standardized Protocol for Urine Collection and Storage recommended by the Human Kidney and Urine Proteome Project (HKUPP), which is specifically designed to ensure compatibility with downstream proteomic analyses.

Mid-stream urine from the second morning void was collected from patients and controls, and stored in sterile urine cups. The samples were then centrifuged at 3000× *g* (4 °C) for 30 min to remove cells and debris. The supernatant was divided into 10 mL aliquots and stored at −80 °C.

### 4.3. Proteomic Analysis

Upon thawing at room temperature, the samples underwent dialysis to remove salts and other interfering substances, using benzoylated dialysis tubing with an average flat width of 32 mm (Sigma-Aldrich, Saint Louis, MO, USA, D7884-10FT, lot: 1003569756). The dialysis tubes were prepared following the protocol provided by Sigma-Aldrich; sulfur compounds were removed by treating with a 0.3% (*w*/*v*) sodium sulfide solution (Sigma-Aldrich, Saint Louis, MO, USA, 407410-10G, lot: 0000130730) at 70 °C for one minute, followed by washing with hot water (60 °C) for two minutes, acidification with 0.2% sulfuric acid, and a final wash with hot water. Each urine aliquot was then transferred into a prepared dialysis tube, which was introduced in a 10 mM PBS solution (Sigma-Aldrich, Saint Louis, MO, USA, P4417-100TAB, lot: 1003393914) at a 1:100 ratio of sample to buffer volume. The tubes were sealed, and overnight dialysis was conducted at room temperature.

The dialyzed samples were concentrated using Vivaspin 6, 5000 MWCO PES, 100 pc (Sartorius Corporation, Bohemia, NY, USA) at 3000× *g* and room temperature to reach a final volume of 500 µL. PIC (Halt Protease Inhibitor cocktail 10 µL, Thermo Fisher Scientific, Waltham, MA, USA) was added to each sample at a 1:100 ratio, followed by drying in SpeedVac. Subsequently, each dried urine sample was dissolved in a Urea Buffer solution (8 M urea, Merck, Darmstadt, Germany, 666122-500GM, lot: 4006230/100 mM Tris, Sigma-Aldrich, Saint Louis, MO, USA, T6791-100G, lot: SLBR4201V). The protein content of every sample was determined using the Bradford Protein Assay (Bio-Rad Laboratories, Hercules, CA, USA, 5000006, lot: 64499411), and the same quantity of total protein amount (50 µg) was used for protein digestion and mass spectrometry analysis.

Protein digestion followed the filter-aided sample preparation (FASP) protocol, a method that combines protein purification and digestion [57]. A total of 50 µg of protein from each sample underwent reduction (using 8 mM DTT in urea buffer—8 M urea and 100 mM Tris), alkylation (with 50 mM IAA in urea buffer, Sigma-Aldrich, Saint Louis, MO, USA, I6125-25G, lot: SLCD4031), and digestion by trypsin on Microcon Centrifugal Filter Devices (Merck Millipore Ltd., Cork, Ireland, PL-10, lot: R1KB26425) at a final concentration of 1 μg/μL.

For bottom-up proteomic analysis, an UltiMate 3000 RSLC nano–HPLC System (Thermo Fisher Scientific) coupled with a high-resolution Orbitrap Fusion Lumos Tribrid Mass Spectrometer (Thermo Fisher Scientific) equipped with an ESI source was used. Peptides were separated on a PepMap RSLC C18 column (2 µM, 100 Å, 50 µm × 15 cm, Thermo Fisher Scientific) using gradient elution. Eluent A consisted of an aqueous solution of 0.1% FA (formic acid), while eluent B was ACN with 0.1% FA. The gradient program was as follows (total runtime: 155 min): 3% B and 97% A (min 0–110), 20% B and 80% A (min 110–120), 40% B and 60% A (min 120–125), 90% B and 10% A (min 125–145), and 3% B and 97% A (min 145–155), with a flow rate of 0.3 μL/min. Each injection volume was 5 μL (containing a total of 1 μg of peptides), with an NSI ion source type, and positive polarity (voltage 1800 V). MS parameters included data-dependent scan mode (DDS) for acquiring high-resolution MS/MS spectra with an Orbitrap detector, a resolution of 120,000 in the 375–1500 *m*/*z* range, and HCD fragmentation. Samples were analyzed in analytical triplicate.

The bottom-up MS/MS data were processed using Proteome Discoverer 2.4.1.15 (2019, Thermo Fisher Scientific) based on the SEQUEST HT algorithm (University of Washington, USA, licensed to Thermo Electron Corp., San Jose, CA, USA) against the Uni-ProtKB/Swiss-Prot *Homo sapiens* database. The parameter settings were as follows: minimum precursor mass, 350 Da; maximum precursor mass, 5000 Da; total intensity threshold, 0.0; minimum peak count, 1; signal-to-noise (S/N) threshold, 1.5; precursor mass tolerance, 10 ppm; fragment mass tolerance, 0.02 Da; use average precursor mass, False; use average fragment mass, False; maximum missed cleavage, 2; minimum peptide length, 6; maximum peptide length, 144. Oxidation/+15.995 Da (M) was set as a dynamic modification, Carbamidomethyl/+57.021 Da (C) as a static modification, with an FDR rate of 0.01 (Strict) and 0.05 (Relaxed). Protein abundance was determined through an LFQ analysis with the following settings: precursor abundance, Area, protein abundance calculation, Top 3 Average. The computational normalization strategy Peptide Total Amount was used during proteins relative quantification, provided by Proteome Discoverer’s quantification workflow.

The results were filtered for high confidence with ≥2 unique peptides (1134 entries). The analytical triplicate mean abundance was considered, and proteins identified and quantified in 50% or more of the samples were selected to build a dataset for further analysis. Contaminants exclusion, differential expression analysis, and statistical validation were conducted using OmicScope (v.1.4.6). A Fold Change (FC) threshold of 1.5 was applied, while the strength of the statistical significance was determined according to the *t*-test (*p*-value cutoff at 0.1, 0.05, and 0.01). FC and the associated *p*-values were calculated using the analytical triplicate–average abundances of proteins (https://omicscope.ib.unicamp.br/OmicScope, accessed on 12 March 2025).

Principal Component Analysis was performed using the MATLAB Statistics and Machine Learning Toolbox (R2024a version, MathWorks Inc., Natick, MA, USA). Pathway enrichment was performed using the Reactome database (http://reactome.org/). Network interactions were illustrated using the Reactome FI (2024 release) plugin for Cytoscape (3.10.1 version). All graphs and heatmaps were generated using an in-house R code and ggplot2, dplyr, tidyr, patchwork, and tidyverse R packages (v. 4.5.1).

The dataset used in this study for the matrisome data analysis was derived from publicly available data provided by MatrisomeDB2.0 (https://matrisomedb.org/). The dataset used for the immune system enrichment was derived from publicly available data provided by the immune cell resource in Human Protein Atlas (https://www.proteinatlas.org/humanproteome/single+cell/immune+cell, accessed on 12 March 2025).

### 4.4. Metabolomic Analysis

A panel of amino acids was investigated. The urine concentrations of amino acids and derivatives were measured using ultraperformance liquid chromatography–MS/MS (UPLC-MS/MS) with the Chromsystems (Chromsystems Instruments & Chemicals GmbH, Gräfelfing, Germany) reagent kit “*MassChrom^®^ Amino Acid Analysis in Urine*” (Chromsystems Instruments & Chemicals GmbH).

Specific gravity was used for the normalization of urinary amino acid concentrations to eliminate bias due to hydration status. Sample preparation and chromatographic separation were performed according to the manufacturer’s kit instructions. The UPLC-MS/MS system consisted of an ExionLC AD UPLC and autosampler system (AB Sciex, Framingham, MA, USA) and a QTRAP 6500+ mass spectrometer (AB Sciex, Framingham, MA, USA) equipped with an electrospray ion source. Analyses were conducted in positive ion mode. The analytical process was monitored using the *MassCheck^®^ Amino Acid Analysis Urine Controls* (Chromsystems Instruments & Chemicals GmbH, Level 1, Level 2, and Level 3, lot: 3622). Urinary amino acid concentrations were determined by comparison with values obtained from a standard curve generated using the *3 PLUS1^®^ Multilevel Urine Calibrator Set* (Chromsystems Instruments & Chemicals GmbH, lot: 4623), manufactured by Chromsystems. For data analysis, including calibration curve generation and amino acid quantification, the SCIEX OS software (v.1.7.0.36606) was used.

AA with >5 missing values were eliminated, while those with missing values < 5 (2.6%) were replaced with LOD, determined as 1/5 of the smallest quantified value for each variable. Then, data were treated with quantile normalization, log-transformation, and autoscaling to perform the bioinformatics analysis. Data were analyzed using Joint Pathways Analysis and differential expression analysis. For the differential expression analysis, FC threshold of 1.2 and *t*-test based *p*-value set at 0.05 were used in MetaboAnalyst 6.0 (https://www.metaboanalyst.ca/, accessed on 20 March 2025). Joint Pathway Analysis was performed using the Targeted Metabolomics option in MetaboAnalyst 6.0, providing a list of metabolites with FC values calculated as the ratio between PEO and Ctrl, and the list of proteins quantified in the samples with corresponding FC values between PEO and Ctrl. The analysis was conducted using the *Homo sapiens* library, and the integrated KEGG Metabolic Pathways database was selected to map both metabolites and proteins onto common Metabolic Pathways.

### 4.5. ATR-FTIR Analysis

For ATR-FTIR, each urine sample before the analysis was left to thaw at room temperature, then vortexed for 1 min. One drop (5 μL) of urine was pipetted onto the surface of UATR (Universal Attenuated Total Reflectance Accessory) diamond. The samples were air-dried on a Diamond/ZnSe crystal and analyzed using a Spectrum Two FTIR spectrophotometer (Perkin Elmer, Norwalk, CT, USA). All FTIR spectra were recorded in the wave number range 4000–450 cm^−1^ with a 4 cm^−1^ resolution. The ATR crystal was cleaned with distilled water and ethanol before every acquisition. Prior to any measurement, the background was collected on diamond and cleaned and it was automatically subtracted from the spectrum of each analyzed sample. Spectra were recorded through a Spectrum v.5.0.1 PerkinElemer software (PerkinElmer, Milan, Italy) in transmittance mode and then were converted to ASCII format. The results were explored using Orange3-3.35.0 and the Orange Spectroscopy add-on for the analysis of spectral data [58]. Spectra underwent a baseline correction and a Standard Normal Variate (SNV) normalization. Then, a *t*-test was performed, using a threshold of 0.5 for the statistical significance and displayed using Excel (Microsoft Excel 365). The Fold Change (1.5 cutoff) for every wavelength was calculated using MetaboAnalyst 6.0.

## 5. Conclusions

Together, these results suggest that PEO is characterized by a complex systemic network involving ECM remodeling, immune activation, hemostatic imbalance, and oxidative–metabolic stress. Importantly, the consistency of GDF15 and CYCS with known mitochondrial biomarkers provides confidence in the validity of these findings.

Given that PEO is a rare and clinically heterogeneous mitochondrial disorder, these results, although derived from a limited patient cohort, highlight reproducible molecular signatures that may support diagnosis, patient stratification, and longitudinal monitoring.

A combined urinary biomarker panel—including stress markers (GDF15, CYCS, and PRDX2), immune effectors (MPO and C4A), ECM proteins (COL1A1 and CCN2), and selected amino acids—could support diagnosis, patient stratification, and longitudinal monitoring in mitochondrial disease.

The statistical power of the study is partly limited by the small sample size, which primarily reflects the rarity of the disorders under investigation and the consequent difficulty in recruiting large, genetically homogeneous cohorts. Nevertheless, in the context of such rare diseases, each individual case represents a highly informative dataset that substantially contributes to elucidating the underlying molecular mechanisms, thereby supporting the overall statistical and biological relevance of our findings.

In conclusion, urinary omics reveal multisystemic dysregulation in PEO and highlight genotype-dependent signatures. These data not only expand the molecular understanding of PEO pathophysiology but also lay the groundwork for the development of non-invasive biomarker panels, with potential applications in personalized medicine and therapeutic monitoring.

## Figures and Tables

**Table 1:** Characteristics of the PEO patients and controls.

	Ctrl	PEO
***Total, n***	8	8
***Age, y (mean ± SD)***	51.4 ± 8.5	49.9 ± 10.5
***Gender (F = female, M = male)***	F = 6, M = 2	F = 6, M = 2
***Variants***		Multiple Deletions = 4 (*POLG* = 2, *TWNK* = 2) mtDNA single deletion = 4

**Table 2:** The table shows the significant results (FDR < 0.05) of the Joint Pathways Analysis using metabolomic and proteomic data. Columns show enriched pathways (Metabolic Pathways), the number of elements known to be in the pathway in KEGG (Total), the number of metabolites/proteins casually expected in every pathway (Expected), the number of metabolites/proteins mapped (Hits), and the statistical significance with Benjamini–Hochberg correction (FDR).

Metabolic Pathways	Total	Expected	Hits	FDR
Aminoacyl-tRNA biosynthesis	74	3.2876	18	8.70 × 10^−8^
Glutathione metabolism	57	2.5323	14	3.25 × 10^−6^
Arginine biosynthesis	28	1.244	10	3.63 × 10^−6^
Glycolysis or gluconeogenesis	60	2.6656	12	1.54 × 10^−4^
Nitrogen metabolism	10	0.44427	5	5.41 × 10^−4^
Glycosaminoglycan degradation	44	1.9548	9	1.26 × 10^−3^
Cysteine and methionine metabolism	72	3.1987	10	1.21 × 10^−2^
Alanine, aspartate, and glutamate metabolism	61	2.71	9	1.23 × 10^−2^
Valine, leucine, and isoleucine biosynthesis	12	0.53312	4	1.25 × 10^−2^
Histidine metabolism	32	1.4217	6	1.91 × 10^−2^

## Data Availability

The original contributions presented in this study are included in the article/Supplementary Materials. Further inquiries can be directed to the corresponding author(s).

## Supplementary Materials

The following supporting information can be downloaded at https://www.mdpi.com/article/10.3390/ijms262311257/s1.
