# Supplementary material for: Urinary Multi-Omics Profiling Reveals Systemic Molecular Alterations in Progressive External Ophthalmoplegia
Source: Int J Mol Sci. 2025 Nov 21;26(23):11257. doi: 10.3390/ijms262311257 (PMC12692649; doi:10.3390/ijms262311257)
Supplement: Supplementary file 1 [file ijms-26-11257-s001.zip › ijms-3957263-supplementary.pdf]

Supplementary Table 1. Pathway enrichment analysis of the proteomic dataset. The table shows the pathways identified from the proteomic analysis, including the Pathway Identifier (unique code associated with each pathway), Pathway Name, Entities Found (number of proteins found in the dataset that map to the pathway), Entities Total (total number of proteins known to be associated with the pathway), Entities Ratio (proportion of identified proteins relative to the total pathway components), Entities pValue (statistical significance of the enrichment), and Entities FDR (false discovery rate-corrected p-value).

**Pathway Identifier**	**Pathway Name**	**Entities Found**	**Entities Total**	**Entities Ratio**	**Entities pValue**	**Entities FDR**
R-HSA-381426	Regulation of Insulin-like Growth Factor (IGF) transport and uptake by Insulin-like Growth Factor Binding Proteins (IGFBPs)	53	124	1.04E-02	1.11E-16	1.60E-14
R-HSA-8957275	Post-translational protein phosphorylation	45	107	9.01E-03	1.11E-16	1.60E-14
R-HSA-6798695	Neutrophil degranulation	129	478	4.02E-02	1.11E-16	1.60E-14
R-HSA-114608	Platelet degranulation	53	128	1.08E-02	1.11E-16	1.60E-14
R-HSA-1474244	Extracellular matrix organization	72	321	2.70E-02	1.11E-16	1.60E-14
R-HSA-76005	Response to elevated platelet cytosolic Ca2+	53	133	1.12E-02	1.11E-16	1.60E-14
R-HSA-76002	Platelet activation, signaling and aggregation	61	265	2.23E-02	1.11E-16	1.60E-14
R-HSA-109582	Hemostasis	108	727	6.12E-02	1.11E-16	1.60E-14
R-HSA-168249	Innate Immune System	189	1187	9.99E-02	1.11E-16	1.60E-14
R-HSA-168256	Immune System	240	2217	1.87E-01	1.11E-16	1.60E-14
R-HSA-216083	Integrin cell surface interactions	31	85	7.16E-03	3.44E-15	4.51E-13
R-HSA-166658	Complement cascade	40	146	1.23E-02	4.11E-15	4.93E-13
R-HSA-977606	Regulation of Complement cascade	38	135	1.14E-02	8.88E-15	9.86E-13
R-HSA-3000178	ECM proteoglycans	27	76	6.40E-03	3.80E-13	3.91E-11
R-HSA-1474228	Degradation of the extracellular matrix	35	140	1.18E-02	2.76E-12	2.65E-10
R-HSA-2173782	Binding and Uptake of Ligands by Scavenger Receptors	32	129	1.09E-02	2.90E-11	2.61E-09
R-HSA-202733	Cell surface interactions at the vascular wall	45	246	2.07E-02	6.84E-11	5.82E-09
R-HSA-5668914	Diseases of metabolism	44	264	2.22E-02	1.85E-09	1.48E-07
R-HSA-2168880	Scavenging of heme from plasma	25	99	8.34E-03	2.97E-09	2.26E-07
R-HSA-140877	Formation of Fibrin Clot (Clotting Cascade)	16	39	3.28E-03	3.21E-09	2.31E-07
R-HSA-198933	Immunoregulatory interactions between a Lymphoid and a non-Lymphoid cell	39	230	1.94E-02	9.94E-09	6.76E-07
R-HSA-2022090	Assembly of collagen fibrils and other multimeric structures	18	61	5.14E-03	5.02E-08	3.26E-06
R-HSA-1442490	Collagen degradation	18	64	5.39E-03	1.01E-07	5.86E-06

R-HSA-166663	Initial triggering of complement	24	111	9.35E-03	1.06E-07	5.86E-06
R-HSA-975634	Retinoid metabolism and transport	15	44	3.70E-03	1.07E-07	5.86E-06
R-HSA-71387	Metabolism of carbohydrates	43	296	2.49E-02	1.29E-07	6.64E-06
R-HSA-3000171	Non-integrin membrane-ECM interactions	20	80	6.74E-03	1.30E-07	6.64E-06
R-HSA-3560782	Diseases associated with glycosaminoglycan metabolism	14	41	3.45E-03	2.76E-07	1.35E-05
R-HSA-420597	Nectin/Necl trans heterodimerization	7	7	5.89E-04	2.85E-07	1.37E-05
R-HSA-6806667	Metabolism of fat-soluble vitamins	15	48	4.04E-03	3.17E-07	1.46E-05
R-HSA-1630316	Glycosaminoglycan metabolism	25	127	1.07E-02	3.24E-07	1.46E-05
R-HSA-418990	Adherens junctions interactions	16	58	4.88E-03	6.58E-07	2.83E-05
R-HSA-1474290	Collagen formation	20	90	7.58E-03	7.96E-07	3.34E-05
R-HSA-140875	Common Pathway of Fibrin Clot Formation	10	22	1.85E-03	1.15E-06	4.71E-05
R-HSA-3781865	Diseases of glycosylation	26	146	1.23E-02	1.20E-06	4.78E-05
R-HSA-140837	Intrinsic Pathway of Fibrin Clot Formation	10	23	1.94E-03	1.70E-06	6.63E-05
R-HSA-8948216	Collagen chain trimerization	13	44	3.70E-03	3.49E-06	1.33E-04
R-HSA-1650814	Collagen biosynthesis and modifying enzymes	16	67	5.64E-03	4.07E-06	1.44E-04
R-HSA-4420332	Defective B3GALT6 causes EDSP2 and SEMDJL1	9	20	1.68E-03	4.25E-06	1.44E-04
R-HSA-3560783	Defective B4GALT7 causes EDS, progeroid type	9	20	1.68E-03	4.25E-06	1.44E-04
R-HSA-3560801	Defective B3GAT3 causes JDSSDHD	9	20	1.68E-03	4.25E-06	1.44E-04
R-HSA-2206281	Mucopolysaccharidoses	7	11	9.26E-04	5.50E-06	1.81E-04
R-HSA-1971475	A tetrasaccharide linker sequence is required for GAG synthesis	10	27	2.27E-03	6.85E-06	2.12E-04
R-HSA-3000170	Syndecan interactions	10	27	2.27E-03	6.85E-06	2.12E-04
R-HSA-5663084	Diseases of carbohydrate metabolism	11	34	2.86E-03	8.46E-06	2.54E-04
R-HSA-1638091	Heparan sulfate/heparin (HS-GAG) metabolism	14	58	4.88E-03	1.42E-05	4.27E-04
R-HSA-1793185	Chondroitin sulfate/dermatan sulfate metabolism	13	51	4.29E-03	1.64E-05	4.70E-04
R-HSA-3000157	Laminin interactions	10	30	2.53E-03	1.68E-05	4.70E-04
R-HSA-8874081	MET activates PTK2 signaling	10	30	2.53E-03	1.68E-05	4.70E-04
R-HSA-2024096	HS-GAG degradation	9	24	2.02E-03	1.78E-05	4.80E-04
R-HSA-166786	Creation of C4 and C2 activators	19	103	8.67E-03	1.98E-05	5.35E-04
R-HSA-173623	Classical antibody-mediated complement activation	18	95	8.00E-03	2.31E-05	6.00E-04
R-HSA-2024101	CS/DS degradation	7	14	1.18E-03	2.55E-05	6.64E-04
R-HSA-5690714	CD22 mediated BCR regulation	15	70	5.89E-03	2.78E-05	6.96E-04
R-HSA-3299685	Detoxification of Reactive Oxygen Species	11	39	3.28E-03	2.94E-05	7.35E-04

R-HSA-1500931	Cell-Cell communication	24	156	1.31E-02	3.20E-05	7.68E-04
R-HSA-6806834	Signaling by MET	16	80	6.74E-03	3.45E-05	8.27E-04
R-HSA-6799990	Metal sequestration by antimicrobial proteins	5	6	5.05E-04	3.49E-05	8.37E-04
R-HSA-446728	Cell junction organization	20	118	9.94E-03	3.91E-05	8.76E-04
R-HSA-2022857	Keratan sulfate degradation	7	15	1.26E-03	3.94E-05	8.76E-04
R-HSA-9734767	Developmental Cell Lineages	16	81	6.82E-03	3.98E-05	8.76E-04
R-HSA-977225	Amyloid fiber formation	16	81	6.82E-03	3.98E-05	8.76E-04
R-HSA-447115	Interleukin-12 family signaling	13	56	4.72E-03	4.23E-05	9.30E-04
R-HSA-421270	Cell-cell junction organization	17	91	7.66E-03	4.57E-05	9.61E-04
R-HSA-2029481	FCGR activation	18	101	8.50E-03	5.02E-05	1.05E-03
R-HSA-8963899	Plasma lipoprotein remodeling	10	35	2.95E-03	6.03E-05	1.27E-03
R-HSA-9613829	Chaperone-Mediated Autophagy	8	22	1.85E-03	6.39E-05	1.28E-03
R-HSA-8862803	Deregulated CDK5 triggers multiple neurodegenerative pathways in Alzheimer's disease models	8	22	1.85E-03	6.39E-05	1.28E-03
R-HSA-8863678	Neurodegenerative Diseases	8	22	1.85E-03	6.39E-05	1.28E-03
R-HSA-174824	Plasma lipoprotein assembly, remodeling, and clearance	15	76	6.40E-03	6.93E-05	1.39E-03
R-HSA-2029485	Role of phospholipids in phagocytosis	19	114	9.60E-03	7.51E-05	1.43E-03
R-HSA-8950505	Gene and protein expression by JAK-STAT signaling after Interleukin-12 stimulation	10	37	3.12E-03	9.47E-05	1.80E-03
R-HSA-2160916	Hyaluronan uptake and degradation	6	12	1.01E-03	9.66E-05	1.84E-03
R-HSA-9020591	Interleukin-12 signaling	11	46	3.87E-03	1.25E-04	2.37E-03
R-HSA-2029482	Regulation of actin dynamics for phagocytic cup formation	22	150	1.26E-02	1.32E-04	2.37E-03
R-HSA-166665	Terminal pathway of complement	5	8	6.74E-04	1.33E-04	2.40E-03
R-HSA-9840310	Glycosphingolipid catabolism	10	39	3.28E-03	1.44E-04	2.60E-03
R-HSA-9734009	Defective Intrinsic Pathway for Apoptosis	8	25	2.11E-03	1.52E-04	2.74E-03
R-HSA-3000480	Scavenging by Class A Receptors	7	19	1.60E-03	1.68E-04	2.86E-03
R-HSA-70263	Gluconeogenesis	8	26	2.19E-03	1.98E-04	3.37E-03
R-HSA-8875878	MET promotes cell motility	10	41	3.45E-03	2.14E-04	3.65E-03
R-HSA-9662851	Anti-inflammatory response favoring Leishmania parasite infection	23	167	1.41E-02	2.28E-04	3.85E-03
R-HSA-9664433	Leishmania parasite growth and survival	23	167	1.41E-02	2.28E-04	3.85E-03
R-HSA-6803157	Antimicrobial peptides	16	95	8.00E-03	2.41E-04	3.85E-03
R-HSA-196854	Metabolism of vitamins and cofactors	26	201	1.69E-02	2.41E-04	3.85E-03
R-HSA-186797	Signaling by PDGF	12	60	5.05E-03	3.18E-04	5.08E-03
R-HSA-1638074	Keratan sulfate/keratin metabolism	9	36	3.03E-03	3.67E-04	5.64E-03

R-HSA-8963888	Chylomicron assembly	5	10	8.42E-04	3.69E-04	5.64E-03
R-HSA-2187338	Visual phototransduction	16	99	8.34E-03	3.76E-04	5.64E-03
R-HSA-2029480	Fcgamma receptor (FCGR) dependent phagocytosis	23	175	1.47E-02	4.34E-04	6.51E-03
R-HSA-2129379	Molecules associated with elastic fibers	9	37	3.12E-03	4.46E-04	6.69E-03
R-HSA-8964058	HDL remodeling	5	11	9.26E-04	5.67E-04	8.50E-03
R-HSA-2022377	Metabolism of Angiotensinogen to Angiotensins	6	17	1.43E-03	6.09E-04	9.05E-03
R-HSA-2142845	Hyaluronan metabolism	6	17	1.43E-03	6.09E-04	9.05E-03
R-HSA-9725554	Differentiation of Keratinocytes in Interfollicular Epidermis in Mammalian Skin	9	39	3.28E-03	6.46E-04	9.05E-03
R-HSA-983695	Antigen activates B Cell Receptor (BCR) leading to generation of second messengers	15	95	8.00E-03	7.13E-04	9.98E-03
R-HSA-9664407	Parasite infection	20	149	1.25E-02	7.76E-04	1.08E-02
R-HSA-9664422	FCGR3A-mediated phagocytosis	20	149	1.25E-02	7.76E-04	1.08E-02
R-HSA-9664417	Leishmania phagocytosis	20	149	1.25E-02	7.76E-04	1.08E-02
R-HSA-2243919	Crosslinking of collagen fibrils	6	18	1.52E-03	8.17E-04	1.08E-02
R-HSA-4341670	Defective NEU1 causes sialidosis	3	3	2.53E-04	8.18E-04	1.08E-02
R-HSA-9664323	FCGR3A-mediated IL10 synthesis	18	128	1.08E-02	8.34E-04	1.08E-02
R-HSA-430116	GP1b-IX-V activation signaling	5	12	1.01E-03	8.35E-04	1.09E-02
R-HSA-1660662	Glycosphingolipid metabolism	11	58	4.88E-03	8.53E-04	1.11E-02
R-HSA-174577	Activation of C3 and C5	4	7	5.89E-04	8.82E-04	1.15E-02
R-HSA-6809371	Formation of the cornified envelope	18	129	1.09E-02	9.09E-04	1.18E-02
R-HSA-5602498	MyD88 deficiency (TLR2/4)	6	19	1.60E-03	1.08E-03	1.40E-02
R-HSA-8963898	Plasma lipoprotein assembly	6	19	1.60E-03	1.08E-03	1.40E-02
R-HSA-9671793	Diseases of hemostasis	6	19	1.60E-03	1.08E-03	1.40E-02
R-HSA-75205	Dissolution of Fibrin Clot	5	13	1.09E-03	1.19E-03	1.42E-02
R-HSA-5603041	IRAK4 deficiency (TLR2/4)	6	20	1.68E-03	1.39E-03	1.67E-02
R-HSA-3595177	Defective CHSY1 causes TPBS	4	8	6.74E-04	1.44E-03	1.72E-02
R-HSA-3595172	Defective CHST3 causes SEDCJD	4	8	6.74E-04	1.44E-03	1.72E-02
R-HSA-3595174	Defective CHST14 causes EDS, musculocontractural type	4	8	6.74E-04	1.44E-03	1.72E-02
R-HSA-1247673	Erythrocytes take up oxygen and release carbon dioxide	4	8	6.74E-04	1.44E-03	1.72E-02
R-HSA-1566948	Elastic fiber formation	9	44	3.70E-03	1.48E-03	1.77E-02
R-HSA-9824443	Parasitic Infection Pathways	28	253	2.13E-02	1.52E-03	1.77E-02
R-HSA-9658195	Leishmania infection	28	253	2.13E-02	1.52E-03	1.77E-02
R-HSA-8963743	Digestion and absorption	7	28	2.36E-03	1.61E-03	1.77E-02

R-HSA-3656237	Defective EXT2 causes exostoses 2	5	14	1.18E-03	1.64E-03	1.80E-02
R-HSA-3656253	Defective EXT1 causes exostoses 1, TRPS2 and CHDS	5	14	1.18E-03	1.64E-03	1.80E-02
R-HSA-163125	Post-translational modification: synthesis of GPI-anchored proteins	14	93	7.83E-03	1.64E-03	1.80E-02
R-HSA-111447	Activation of BAD and translocation to mitochondria	5	15	1.26E-03	2.21E-03	2.43E-02
R-HSA-5686938	Regulation of TLR by endogenous ligand	6	22	1.85E-03	2.24E-03	2.46E-02
R-HSA-8935690	Digestion	6	23	1.94E-03	2.78E-03	3.06E-02
R-HSA-75892	Platelet Adhesion to exposed collagen	5	16	1.35E-03	2.90E-03	3.19E-02
R-HSA-8963901	Chylomicron remodeling	4	10	8.42E-04	3.20E-03	3.52E-02
R-HSA-173736	Alternative complement activation	3	5	4.21E-04	3.47E-03	3.71E-02
R-HSA-8852405	Signaling by MST1	3	5	4.21E-04	3.47E-03	3.71E-02
R-HSA-2730905	Role of LAT2/NTAL/LAB on calcium mobilization	14	102	8.59E-03	3.71E-03	3.71E-02
R-HSA-5694530	Cargo concentration in the ER	7	33	2.78E-03	3.97E-03	3.97E-02
R-HSA-419037	NCAM1 interactions	8	42	3.54E-03	4.07E-03	4.07E-02
R-HSA-9694614	Attachment and Entry	6	25	2.11E-03	4.16E-03	4.16E-02
R-HSA-2022923	Dermatan sulfate biosynthesis	4	11	9.26E-04	4.47E-03	4.47E-02
R-HSA-189085	Digestion of dietary carbohydrate	4	11	9.26E-04	4.47E-03	4.47E-02
R-HSA-9925561	Developmental Lineage of Pancreatic Acinar Cells	8	43	3.62E-03	4.67E-03	4.67E-02

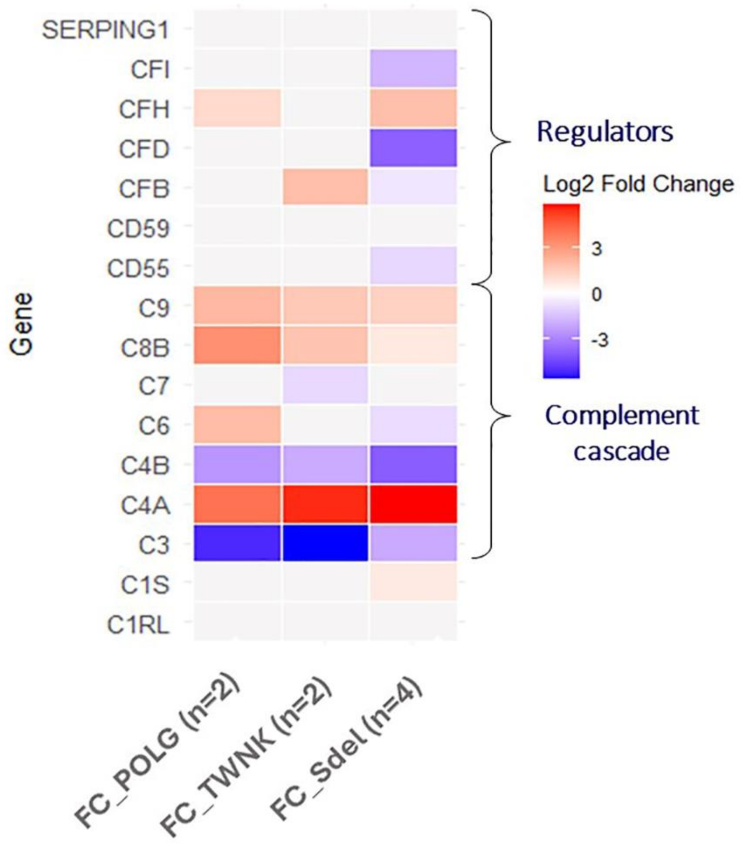

Supplementary Figure 1. The POLG, TWNK, and Sdel variants' contribution to the complement cascade and complement cascade regulator enzymes. Heatmaps display the Log2FC values (x-axis) for the POLG *vs.* Ctrl, TWNK *vs.* Ctrl, and Sdel *vs.* Ctrl ratios. A Fold Change (FC) threshold of 1.5 was applied. Proteins upregulated in the variants with respect to Ctrl are shown in red, while the downregulated ones are in blue.

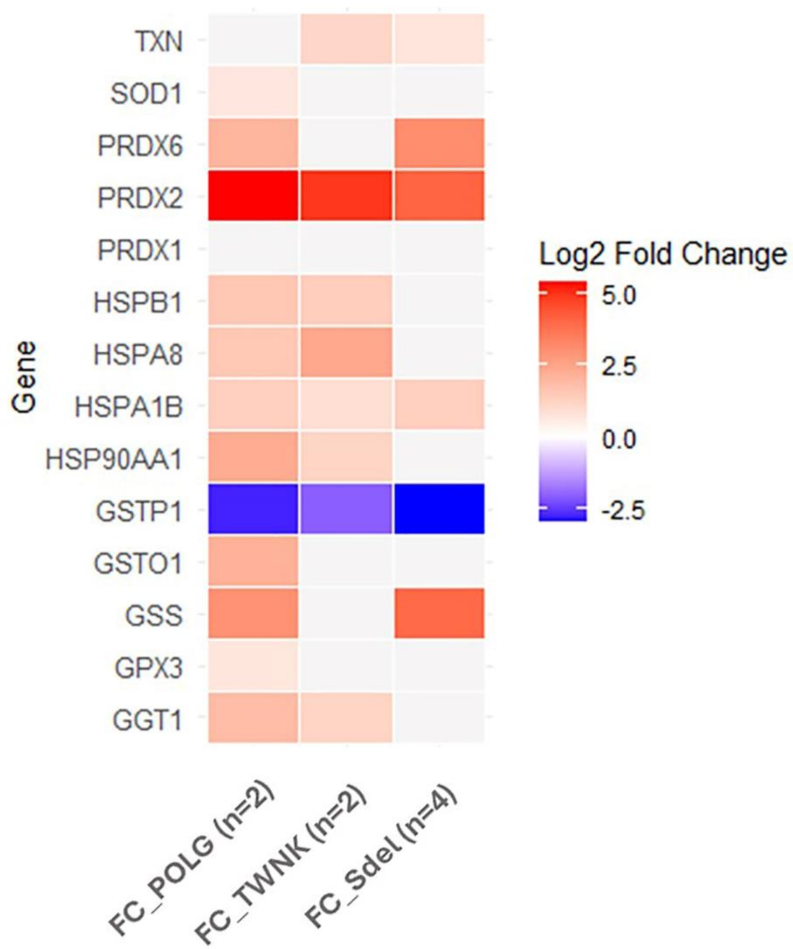

Supplementary Figure 2. The POLG, TWNK, and Sdel variants' contribution to the oxidative stress response. Heatmaps display the Log2FC values (x-axis) for the POLG *vs.* Ctrl, TWNK *vs.* Ctrl and Sdel *vs.* Ctrl ratios. A Fold Change (FC) threshold of 1.5 was applied. Proteins upregulated in the variants with respect to Ctrl are shown in red, while the downregulated ones are in blue.

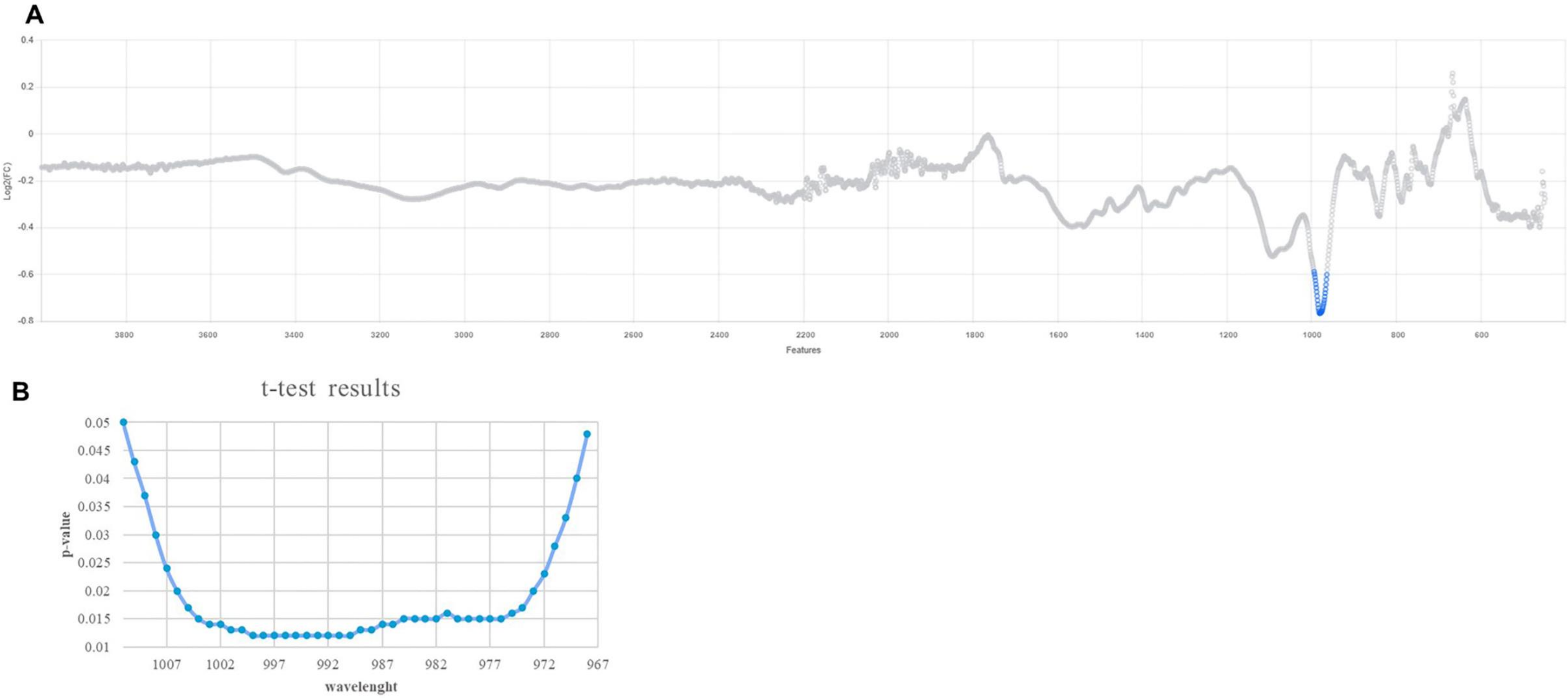

Supplementary Figure 3. Representative ratio FTIR spectra and statistical t-test analysis of urinary samples from PEO patients and Ctrl. A The plot shows the PEO vs. Ctrl absorbance ratio after SNV normalization (y-axis) across the 4000–450 cm^{-1} spectral range (x-axis). Wavenumbers with a ratio >|1.5| are highlighted in blue and correspond to the 1011–967 cm^{-1} region. **B** The plot reports the results of t-tests performed on each wavenumber within this region (x-axis), showing significant differences ($p < 0.05$, y-axis) between groups.
